# Literary Notes

**Published:** 1906-12

**Authors:** 


### LITERARY NOTES.

THE Therapeutic Gazette, the Medical Age and Medicine will be consolidated January 1, 1907, and become one magazine. The publication will be edited by Drs. Hobart A. Hare and Edward Martin, who have been editors of the Therapeutic Gazette for several years. The title of the consolidated magazine will be The Therapeutic Gazette, incorporating Medicine and the Medical Age. The subscription price will be $2.00 a year. E. G. Swift is the publisher and Harry Skillman is the business manager.

ST. LOUIS *Medical and Surgical Journal* has combined with the *Medical Mirror*. The former journal is absorbed in the latter.

